# Genome-wide characterization of the *abscisic acid*-, *stress*- and *ripening*-*induced* (*ASR*) gene family in wheat (*Triticum aestivum* L.)

**DOI:** 10.1186/s40659-020-00291-6

**Published:** 2020-05-24

**Authors:** Huawei Li, Haiying Guan, Qicui Zhuo, Zongshuai Wang, Shengdong Li, Jisheng Si, Bin Zhang, Bo Feng, Ling-an Kong, Fahong Wang, Zheng Wang, Lishun Zhang

**Affiliations:** 1 Crop Research Institute, Shandong Academy of Agricultural Sciences, 202 Gongyebei Road, Jinan, 250100 China; 2 Maize Research Institute, Shandong Academy of Agricultural Sciences/National Engineering Laboratory of Wheat and Maize/Key Laboratory of Biology and Genetic Improvement of Maize in Northern Yellow-Huai Rivers Plain, Ministry of Agriculture, Jinan, 250100 Shandong China; 3 Jinan Yongfeng Seed Industry Co., Ltd, 3620 Pingannan Road, Jinan, 250100 China

**Keywords:** Abscisic acid-, stress-, and ripening-induced (ASR), Genome-wide, Tandem and segmental duplication, Phylogenetic analyses, Gene structure, Salt

## Abstract

**Background:**

*Abscisic acid*-, *stress*-, and *ripening*-*induced* (*ASR*) genes are a class of plant specific transcription factors (TFs), which play important roles in plant development, growth and abiotic stress responses. The wheat *ASRs* have not been described in genome-wide yet.

**Methods:**

We predicted the transmembrane regions and subcellular localization using the TMHMM server, and Plant-mPLoc server and CELLO v2.5, respectively. Then the phylogeny tree was built by MEGA7. The exon–intron structures, conserved motifs and TFs binding sites were analyzed by GSDS, MEME program and PlantRegMap, respectively.

**Results:**

In wheat, 33*ASR* genes were identified through a genome-wide survey and classified into six groups. Phylogenetic analyses revealed that the TaASR proteins in the same group tightly clustered together, compared with those from other species. Duplication analysis indicated that the *TaASR* gene family has expanded mainly through tandem and segmental duplication events. Similar gene structures and conserved protein motifs of TaASRs in wheat were identified in the same groups. *ASR* genes contained various TF binding cites associated with the stress responses in the promoter region. Gene expression was generally associated with the expected group-specific expression pattern in five tissues, including grain, leaf, root, spike and stem, indicating the broad conservation of *ASR* genes function during wheat evolution. The qRT-PCR analysis revealed that several *ASRs* were up-regulated in response to NaCl and PEG stress.

**Conclusion:**

We identified *ASR* genes in wheat and found that gene duplication events are the main driving force for *ASR* gene evolution in wheat. The expression of wheat *ASR* genes was modulated in responses to multiple abiotic stresses, including drought/osmotic and salt stress. The results provided important information for further identifications of the functions of wheat *ASR* genes and candidate genes for high abiotic stress tolerant wheat breeding.

## Background

ASR is a kind of plant specific, small and hydrophilic protein. As the first member of *ASR* gene family, *ASR1* was identified by differential screening a tomato (*Solanum lycopersicum* L.) fruit cDNA library with cDNA from stressed leaves [1]. Then, a large number of *ASR* homologs were detected from a wide range of other plant species, including gymnosperms, (e.g., loblolly pine (*Pinustaeda* L.) and ginkgo (*Ginkgo biloba* L.)) [2–4], monocots, (e.g., rice (*Oryza sativa* L.), maize (*Zea mays* L.) and *Brachypodium distachyon*) [5–9], and dicots (e.g., soybean (*Glycine max*), common bean (*Phaseolus vulgaris* L.) and potato (*Solanum tuberosum* L.)) [10–12]. Interestingly, they are missing in the model plant *Arabidopsis thaliana* [13]. The number of *ASR* members differs among plant species, none for *Arabidopsis thaliana* [13, 14], four for loblolly pine [2, 3], five for tomato and *Brachypodium distachyon* [8, 15], six for rice and foxtail millet [5, 16], and ten for maize [9]. Two types of events can induce extension of gene families: (1) whole genome duplication (WGD) [17] and (2) small-scale gene duplication, including tandem and segmental duplication [18].

Some ASR proteins, such as ASR1 (VvMSA) in grape, NtTIP1 (orthology of ASR2 in tomato) in tobacco, and ASR1 in sorghum and wheat, are located in the nucleus and functioning as transcription factors [19–22]. Other ASR proteins such as ASR1 in tomato, ASR1 and ASR5 in rice, ASR1 to ASR5 (except ASR3) in maize, are located both in the nucleus and the cytosol [9, 23, 24]. Increasing evidences showed that ASR proteins play important roles in plant growth and fruit ripening [1, 25–27], as well as in regulation of floral development and flowering time [15, 28, 29]. The ASR proteins have been well documented for their responses to multiple abiotic stresses, including drought, salt, heat, cold, and exposure to cadmium (Cd) and aluminum (Al) [9, 24, 30–38].

Several studies indicated the positive roles of plant *ASRs* in adaption to abiotic stresses at transcriptional level. Overexpression of the *LLA23* gene from lily (*Lilium longiflorum*) in *Arabidopsis* enhanced drought tolerance via up regulating the expression of ABA/stress-regulated genes [14, 28]. Also, it has been documented that overexpression of *LLA23* gene in *Arabidopsis* conferred the cold and freezing tolerance [39]. Likewise, overexpression plantain *MpASR* and maize *ZmASR3* in *Arabidopsis*, tomato *SlASR1*, *Brachypodium distachyonBdASR1* and wheat *ASR1* in tobacco all improved the tolerance to water stress [8, 21, 37, 40–42]. In addition, transgenic rice plants with overexpression of *OsASR2* enhanced the tolerance to drought by targeting the GT-1 cis-element [35], while overexpression of *OsASR5* enhanced the drought tolerance by regulating ABA synthesis, promoting stomatal closure, and acting as chaperone-like protein [34]. It was also illuminated that *OsASR5* is involved in response to aluminum (Al) stress in rice [24]. The transgenic rice with overexpression of *OsASR1* and transgenic maize with overexpression of *ZmASR1* increased their cold tolerance [36, 43]. Transgenic *Arabidopsis* plants with overexpression of ipomoea pes-caprae *IpASR* and oxtail millet *SiASR4* both enhanced the tolerance to salt stress [33, 44].

In the previous study, one member (i.e. *TaASR4D* here) of the ASR family was characterized in wheat [21]. Nevertheless, a comprehensive characterization of the *ASR* family in wheat has not been developed. The draft genome of “Chinese Spring” bread wheat has been completed by various sequencing technologies [45–47]. In addition, the physical map (IWGSC, 2018) and a high-quality genome have been published [48], allowing the isolation and analysis of gene families in the genome-wide in wheat. In this study, a total of 33 *ASR* members were isolated in wheat, and the sequence characteristics, chromosomal distribution and duplication, phylogenetic relationship, gene structure and conserved motif and TF binding sites were analyzed. The tissue specific expression and expression profiles under various abiotic stresses were also examined using the public RNA-seq data and quantitative real-time-PCR (qRT-PCR). These results will provide a better understanding of the wheat *ASR* family members and important information for subsequent studies and utilization of *TaASRs* in wheat.

## Methods

### Genome-wide identification of ASR gene family in wheat

*ASR* genes reported in other species such as apple and rice were retrieved and downloaded [5, 49]. Their amino acid sequences were used to construct a hidden Markov model (HMM) profile of ASR using the hmmbuild procedure (HMMER3.0) (http://hmmer.org) [50]. The dataset of wheat proteins (https://urgi.versailles.inra.fr/download/iwgsc/IWGSC_RefSeq_Assemblies/v1.0) were searched using BLASTP with the HMM profile of ASR as a query and all possible ASR protein sequences were extracted (e-value ≤ 1e^−10^). The self-BLASTP search was first used to remove the redundant sequences among them (e-value ≤ 1e^−10^). Subsequently, the PFAM (http://pfam.xfam.org/) and SMART (http://smart.emblheidelberg.de/) website were used to confirm all the TaASRs containing the abscisic acid (ABA)/water deficit stress (WDS) domain (PF02496.15). The features of each protein, such as the numbers of amino acids, molecular weight (Da), isoelectric point (pI) and gravity, were calculated using ExPASy (https://web.expasy.org/protparam/) [51]. The trans-membrane structure was obtained using TMHMM Server 2.0 online tool (http://www.cbs.dtu.dk/services/TMHMM/). The subcellular localization of each TaASR was predicted using the online tools Plant-mPLoc server (http://www.csbio.sjtu.edu.cn/bioinf/plant-multi) and CELLO v2.5 (http://cello.life.nctu.edu.tw).

### Phylogenetic analysis

The amino acid sequences of the ASR proteins from wheat and other 7 species including *Brachypodium distachyon*, common bean, foxtail millet, maize, rice, sorghum and soybean were downloaded from the URGI database (https://urgi.versailles.inra.fr/download/iwgsc/IWGSC_RefSeq_Assemblies/v1.0/) and JGI Phytozome (https://phytozome.jgi.doe.gov/pz/portal.html), respectively. These protein sequences and ID loci are listed in Additional file 1: Table S1. To compare the evolutionary relationships among these ASR proteins, the amino acid sequences were aligned using the ClustalW program implemented in MEGA7.0 (http://www.megasoftware.net/). The phylogenetic tree was constructed by using the neighbor-joining (NJ) method based on the JTT matrix-based model with 1000 bootstrap replications [52]. Another phylogenetic tree was constructed using the protein sequences from wheat *ASR* gene family to understand the evolution of its own members.

### Chromosomal locations and gene duplication

The chromosomal localization of each *TaASR* gene was analyzed by mapping its sequence back to the corresponding chromosome of wheat (IWGSC RefSeq v1.0) using BLAST program with the E-value < 10^−5^. To detect the gene homology, the protein sequences of *ASR* genes in wheat were blasted against each other by BLASTP (E value < 10^−20^, identity > 75%) [53, 54]. Tandem duplicated *TaASR* genes were defined as two or more adjacent homologous genes located on a single chromosome within 150 kb without any intervening gene, while homologous genes among different chromosomes were defined as segmental duplicated genes [18, 55]. The chromosomal distribution and synteny of these *ASR* genes was visualized by the CIRCOS program [56].

### Gene structure and conserved motif analysis

To predict the exon–intron structures of the wheat *ASR* genes, GSDS (http://gsds.cbi.pku.edu.cn/) was used by comparing the coding/cDNA sequence with its genomic sequence of each gene. To identify the conserved motifs, the MEME program (http://meme-suite.org/) was used with the following parameters: the optimum motif widths of 6–50 amino acid residues and the maximum number of 20 motifs.

### Transcription factor binding sites predication

To identify the binding sites of transcription factors in the promoter region of each *TaASR* gene, 2000-bp genomic DNA sequence upstream of the transcriptional start site used as the promoter sequence was searched via the database PlantRegMap (http://plantregmap.cbi.pku.edu.cn/binding_site_prediction.php) with the following parameters: e-value ≤ 1e^−15^ and the top number of 12.

### Expression profile analysis of TaASR genes by RNA-seq data

RNA-seq data of five tissues each at three different developmental stages (grain at Z71, Z75, Z85; leaf at Z10, Z23, Z71; root at Z10, Z13, Z39; spike at Z32, Z39, Z65; stem at Z30, Z32, Z65) in bread wheat c.v. Chinese spring with study title “choulet_URGI” was retrieved from expVIP (http://www.wheat-expression.com/), and then the log2 (FPKM + 1) (FPKM, fragments per kilobase transcript per million reads mapped) value of each *TaASR* was used for visualizing the heat map as a green-yellow–red gradient. The heat map was generated by using the pheatmap package in Rversion 3.5.2 (https://www.r-project.org/).

### Plant growth, stress treatment and qRT-PCR

Wheat ‘c.v. JM262’ seeds were grown in a growth chamber under controlled conditions as Hu et al. [57] described. For abiotic stress experiments, 15-day-old wheat seedlings were exposed to salt stress (200 mM NaCl solution for 6 and 24 h) and drought stress (23% (w/v) PEG-6000 solution for 6 and 48 h) as described previously [57, 58]. All the treatments were performed with three biological replications. Seedlings grown under the non-stress condition were used as the control. Leaves and roots were collected from ten plants at the above-mentioned time points under both stress and non-stress conditions. Samples were immediately frozen in liquid nitrogen and stored at − 80 °C for further analysis.

Total RNA from all samples was isolated using TRIzol Reagent (Invitrogen Corp., Carlsbad, CA) according to the manufacturer’s instructions. DNase I was used to eliminate the genomic DNA contamination. Then, the first strand cDNA was synthesized with oligodT primer using the Prime Script II kit (TaKaRa, Dalian). Finally, qRT-PCR was performed in a 20 μl reaction volume using SYBR Green PCR master mix (TaKaRa, Dalian) on ABI 7500 Real-time PCR system (Applied Biosystems, USA), and three technical replicates were conducted for each reaction. The PCR processes were as follows: 95 °C for 30 s, 40 cycles of 95 °C for 3 s and 60 °C for 30 s, followed by a melting curve analysis of 95 °C for 15 s, 60 °C for 60 s, and 95 °C for 15 s. For relative quantification, the 2^−ΔΔCT^method was used [59], with wheat *actin* gene used as an internal reference. The quantitative primers were designed using Primer 5.0 and listed in Additional file 2: Table S2.

## Results

### Identification and characterization of the ASR gene family in wheat

The *TaASR4D* was previously cloned and characterized [21]; however, the information of other *ASR* family members is rarely gained in wheat. The recently released genome database (IWGSC RefSeq v1.0) shed light on the possibility to identify other *ASR* family members in wheat. A total of 33 non-redundant wheat ASRs containing the complete ABA/WDS domain were retrieved based on a genome-wide search and confirmation (Table 1). Since there is no standard nomenclature, they were named based on their original sequence ID and their homologous relationships, and designated as *TaASR1D*-*10A6* (Table 1). In these *TaASRs*, the ORF length ranged from 840 (*TaASR5B*) to 231 (*TaASR6B*) bp, with an average size of 465 bp, and the protein length ranged from 279 (*TaASR5B*) to 76 (*TaASR6B*) amino acids (aa), with an average size of 154 aa. The predicted molecular weight (Mw), isoelectric point (pI) and gravity ranged from 30.34 (*TaASR5B*) to 8.65 kDa (*TaASR6B*), 10.14 (*TaASR9D*) to 4.97 (*TaASR5B*) and − 0.917 (*TaASR2D*) to − 1.760 (*TaASR5B*), and the average size is 16.84 kDa, 8.05 and − 1.226, respectively. The predicted transmembrane structure analysis revealed that all examined TaASRs had no transmembrane segment. The theoretical subcellular localization analysis showed that all 33 TaASRs are localized in the nucleus based on the consistent results predicted using Plant-mPLoc and CELLO v2.5 subcellular localization prediction software.

**Table 1:** Characteristics of the ASR gene family members in wheat

Gene name	ID	Chromosome localtion	Exon number	ORF (bp)	AA (aa)	Mw (kDa)	PI	Gravity	Subcellular location
*TaASR1D*	TraesCS3D01G517400.1	chr3D: 600607844-600608597	2	663	220	23.26	6.19	− 0.986	Nuclear
*TaASR1B*	TraesCS3B01G578500.1	chr3B: 807930370-807931125	2	660	219	23.14	6.03	− 0.955	Nuclear
*TaASR1A*	TraesCS3A01G509800.1	chr3A: 730177607-730178363	2	660	219	23.18	6.25	− 0.977	Nuclear
*TaASR2A*	TraesCS3A01G509900.1	chr3A: 730207889-730208652	2	657	218	23.21	6.24	− 0.929	Nuclear
*TaASR2D*	TraesCS3D01G517300.1	chr3D: 600594427-600595179	2	657	218	23.20	6.24	− 0.917	Nuclear
*TaASR2B*	TraesCS3B01G578400.1	chr3B: 807881862-807882916	2	666	221	23.45	6.10	− 0.950	Nuclear
*TaASR3A1*	TraesCS3A01G510100.1	chr3A: 730279210-730279978	2	660	219	23.68	6.16	− 1.074	Nuclear
*TaASR3D*	TraesCS3D01G517100.1	chr3D: 600563453-600564143	3	528	175	18.86	6.51	− 1.106	Nuclear
*TaASR3B*	TraesCS3B01G578200.1	chr3B: 807812908-807813694	2	684	227	24.39	6.27	− 1.087	Nuclear
*TaASR3A2*	TraesCS3A01G510200.1	chr3A: 730327845-730328798	2	693	230	24.91	7.79	− 0.991	Nuclear
*TaASR4D*	TraesCS4D01G109500.1	chr4D: 88700513-88701275	2	414	137	15.30	6.06	− 1.198	Nuclear
*TaASR4B*	TraesCS4B01G112000.1	chr4B: 125481409-125482171	2	417	138	15.46	6.14	− 1.199	Nuclear
*TaASR4A*	TraesCS4A01G208400.1	chr4A: 501468566-501469147	3	405	134	15.00	6.11	− 1.043	Nuclear
*TaASR5A*	TraesCS2A01G301500.1	chr2A: 516531874-516532791	2	795	264	28.83	5.19	− 1.755	Nuclear
*TaASR5B*	TraesCS2B01G317600.1	chr2B: 453077970-453078937	2	840	279	30.34	4.97	− 1.760	Nuclear
*TaASR5D*	TraesCS2D01G300100.1	chr2D: 382195603-382196510	2	789	262	28.65	5.20	− 1.735	Nuclear
*TaASR6D*	TraesCS3D01G517700.1	chr3D: 600624824-600625221	2	285	94	10.37	9.74	− 1.233	Nuclear
*TaASR6A*	TraesCS3A01G509700.1	chr3A: 730152160-730152564	2	285	94	10.40	9.82	− 1.234	Nuclear
*TaASR6B*	TraesCS3B01G858500LC.1	chr3B: 807959304-807959643	2	231	76	8.65	9.65	− 1.261	Nuclear
*TaASR7D1*	TraesCS3D01G517500.1	chr3D: 600617363-600617756	2	285	94	10.44	9.70	− 1.219	Nuclear
*TaASR7D2*	TraesCS3D01G517600.1	chr3D: 600621726-600622110	2	276	91	10.10	9.87	− 1.209	Nuclear
*TaASR8B*	TraesCS3B01G578800.1	chr3B: 808019716-808020116	2	294	97	10.84	9.99	− 1.332	Nuclear
*TaASR8D*	TraesCS3D01G518000.1	chr3D: 600655220-600655626	2	294	97	10.81	10.04	− 1.366	Nuclear
*TaASR9A*	TraesCS3A01G509400.1	chr3A: 730085763-730086163	2	294	97	10.76	9.99	− 1.331	Nuclear
*TaASR9B*	TraesCS3B01G456200.1	chr3B: 697683251-697683662	2	303	100	11.04	9.99	− 1.349	Nuclear
*TaASR9D*	TraesCS3D01G518100.1	chr3D: 600665541-600665933	2	303	100	11.16	10.14	− 1.325	Nuclear
*TaASR10A1*	TraesCS3A01G692700LC.1	chr3A: 730031423-730032153	2	333	110	12.34	9.89	− 1.286	Nuclear
*TaASR10A2*	TraesCS3A01G510700.1	chr3A: 730463302-730463736	2	333	110	12.34	9.89	− 1.286	Nuclear
*TaASR10U*	TraesCSU01G240200.1	chrUn: 358203209-358203643	2	333	110	12.34	9.89	− 1.286	Nuclear
*TaASR10A3*	TraesCS3A01G693700LC.1	chr3A: 730442877-730443311	2	333	110	12.34	9.89	− 1.286	Nuclear
*TaASR10A4*	TraesCS3A01G693800LC.1	chr3A: 730591083-730591517	2	333	110	12.34	9.89	− 1.286	Nuclear
*TaASR10A5*	TraesCS3A01G509200.1	chr3A: 730011752-730012186	2	333	110	12.34	9.89	− 1.286	Nuclear
*TaASR10A6*	TraesCS3A01G509500.1	chr3A: 730092027-730092461	2	333	110	12.34	9.89	− 1.286	Nuclear

ORF indicates open reading frame, AA indicates amino acids, Mw indicates protein molecular weight, and PI indicates protein isoelectric point

### Phylogenetic analysis of ASR genes

**Fig. 1:**
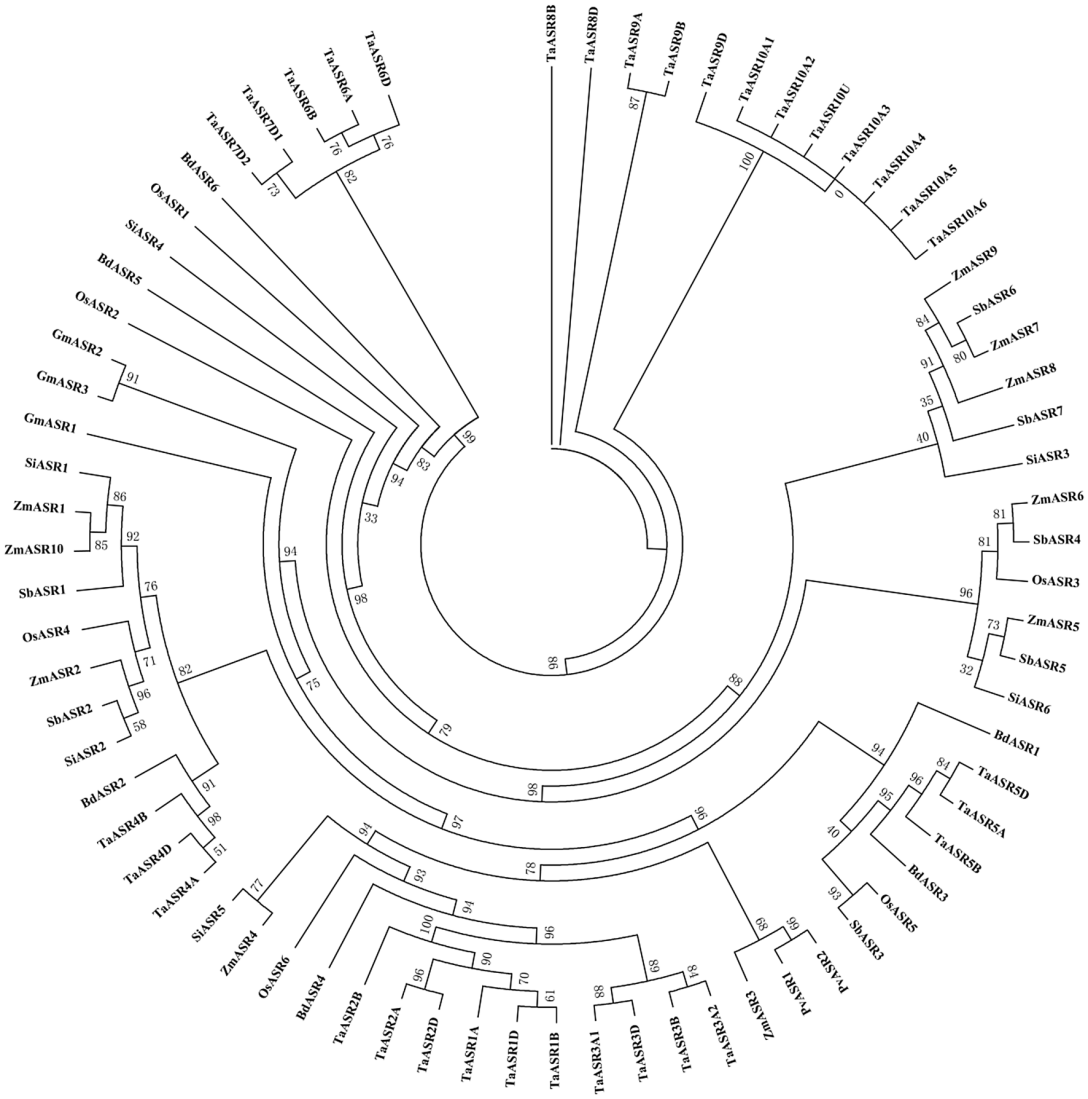
Phylogenetic analysis of ASR proteins among wheat (33), maize (10), sorghum (7), rice (6), foxtail millet (6), *Brachypodium distachyon* (6), soybean (3) and common bean (2)

To compare the evolutionary relationships among these *TaASR* genes, the phylogenetic tree of TaASRs was constructed using the full length protein sequences (Additional file 3: Figure S1). The 33 TaASR proteins could be clustered into six groups with uneven numbers of family members, 10 in group I (TaASR1D-3A2) representing the largest group of ASRs, 3 in group II (TaASR4D-4A) and III (TaASR5A-5D), 5 in group IV (TaASR6D-7D2) and V (TaASR8B-9D), and 7 in group VI (TaASR10A1-10A6), respectively. To further compare the evolutionary relationships of ASR proteins, a phylogenetic tree was constructed using the protein sequences of *ASR* genes from wheat (33), *Brachypodium distachyon* (6), common bean (2), foxtail millet (6), maize (10), rice (6), sorghum (7) and soybean (3) (Fig. 1, Additional file 1: Table S1). The wheat ASR proteins in each group (except TaASR9D in group V) were tightly clustered together rather than with ASR proteins from other species, especially those in group IV, V and VI. In addition, wheat group I was clustered with BdASR4, OsASR6, ZmASR4, SiASR5, ZmASR3, PvASR1 and PvASR2. Wheat group II was related to BdASR2, SiASR2, SbASR2, ZmASR2, OsASR4, SbASR1, ZmASR10, ZmASR1 and SiASR1. However, wheat group III was clustered with BdASR3, OsASR5, SbASR3 and BdASR1. It was indicated that the wheat ASR proteins share high homology with the ASR proteins of other plants (Fig. 1).

#### Chromosomal distribution and gene duplication patterns of wheat ASR genes

**Fig. 2:**
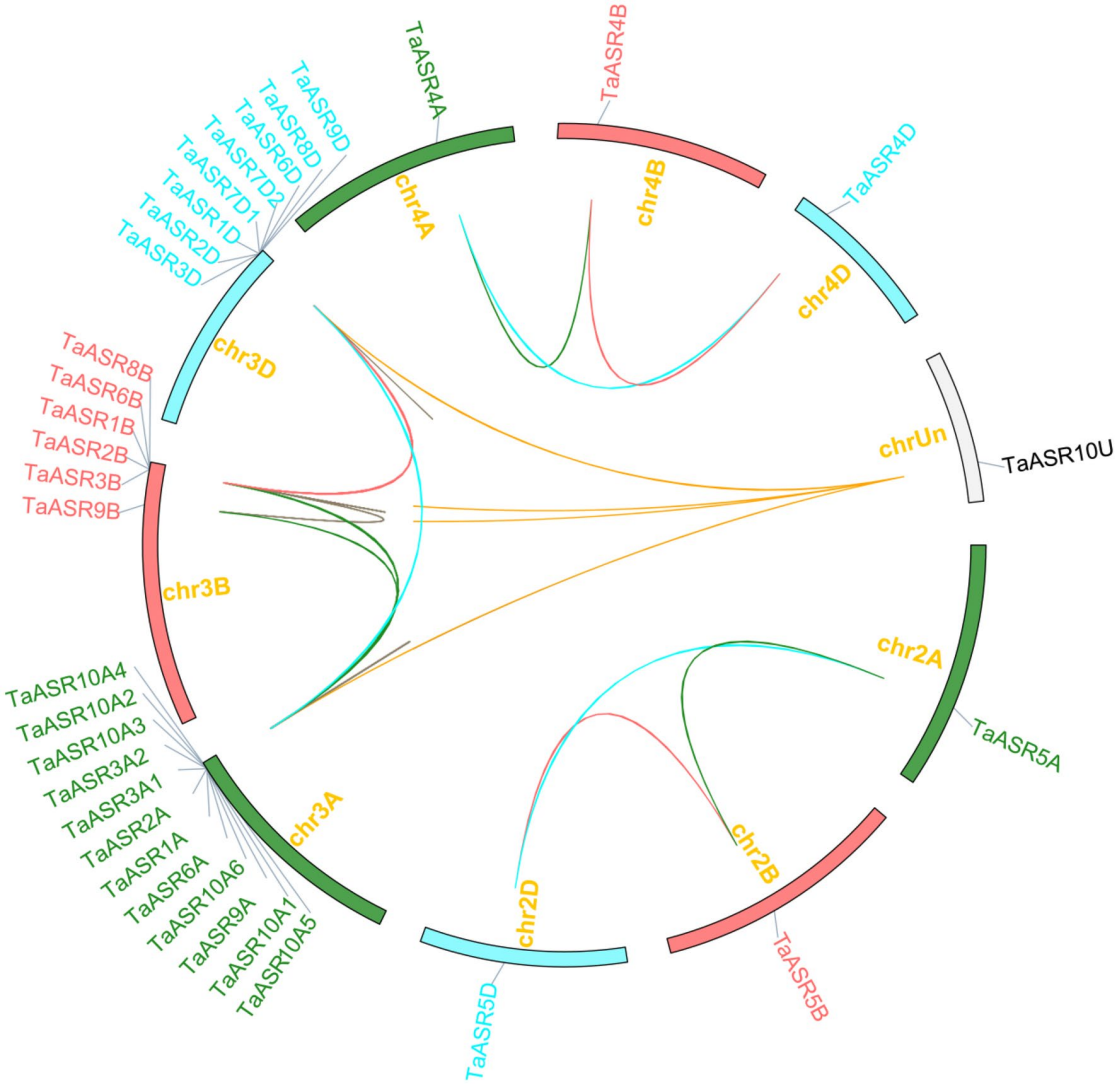
Chromosomal distribution of *TaASR* genes and gene duplication analysis in wheat. Different color lines indicate duplicated *ASR* gene pairs on different chromosome

Chromosomal distribution analysis showed that, except for *TaASR10U* located on the unanchored scaffolds, 32 *TaASR* genes were unevenly mapped on 9 of 21 wheat chromosomes (Table 1, Fig. 2). A total of 12, 6 and 8 *TaASR* genes were located on the distal of chromosomes 3A, 3B and 3D, which represented 36.4%, 18.2% and 24.2% of total, respectively. Besides, one gene (3.0%) was located on chromosomes 2A, 2B, 2D, 4A, 4B and 4D, respectively. In contrast, no one was located on the remaining 12 chromosomes. The *TaASR* genes were unevenly distributed among the sub-genomes A, B, and D, with 14, 8 and 10 members, representing 42.4%, 24.2% and 30.3% of total, respectively. Moreover, wheat *ASR* genes were unevenly distributed among different chromosomal groups. The chromosomal group III carried 26 *TaASR* genes (78.8%), representing the largest number, followed by the groups II and IV, which carried 3 genes (9.0%). The rest four chromosomal groups including I, V, VI and VII carried no *TaASR* gene. Furthermore, most of these genes were tightly linked and lay within clusters. For example, 12, 5 (except *TaASR9B*) and 8 *TaASR* genes were close on chromosome 3A, 3B and 3D within 579.8, 207.2 and 102.5 kb, respectively. Interestingly, all seven group VI members except *TaASR10U* were tightly linked and lay on the distal of chromosome 3A. Given that the protein sequence of *TaASR10U* was same with its closed related six members (*TaASR10A1, TaASR10A2*, *TaASR10A3*, *TaASR10A4*, *TaASR10A5* and *TaASR10A6*), we speculated that *TaASR10U* might be linked with them and it was located on the distal of chromosome 3A.

Tandem and segmental duplications are essential for gene family evolution to generate new gene members [60, 61]. Thus, we analyzed the duplication events of wheat *ASR* genes. A total of 14 pairs of genes which corresponded to 23 wheat *ASR* genes were identified as tandem duplication genes and located on chromosome 3 (A, B, D) (Additional file 4: Table S3, Fig. 2, Table 1). Additionally, one group of 5 tandem duplicated genes were located on chromosome 3A within 140.8 kb, two groups of 3 tandem duplicated genes were located on chromosomes 3A and 3D within 148.6 and 7.9 kb, and two groups of 2 tandem duplicated genes were located on chromosomes 3A, 3B and 3D, within 31.0/49.6 kb, 49.3/60.8 kb, and 14.2/10.7 kb, respectively. Furthermore, eight homoeologous gene groups (24 *TaASR* genes) might be related to segmental duplication events, which were distributed on chromosomes 2 (A, B, D), 3 (A, B, D) and 4 (A, B, D) (Additional file 4: Table S3, Figs. 1, 2, Table 1). These results showed that there is a high degree of homology between the homologous chromosomes. Interestingly, not every *ASR* had three homoeologous genes on the homologous chromosomes 3A, 3B, and 3D. 1 pair of *TaASRs* only had two homoeologous genes (*TaASR8B*, *TaASR8D*) on the homologous chromosomes 3B and 3D. Another 1 pair of *TaASRs* had two genes (*TaASR3A1*, *TaASR3A2*) on the homologous chromosomes 3A. Additionally, *TaASR7* repeated once (*TaASR7D1*/*7D2*) only on the chromosome 3D, *TaASR10* repeated 6 times (*TaASR10A1*-*A6*) only on the chromosome 3A, and one gene (*TaASR10U*) distributed on the unanchored scaffolds. These results indicate that there might be independent evolution and repetitive events between the homologous chromosomes. There was still 1 *TaASRs* (*TaASR10U*) which was neither tandem nor segmental duplication gene. This result indicated that the tandem and segmental duplication events were essential for the expansion of the wheat *ASR* gene family.

### Gene structure and conserved motifs of ASR genes in wheat

**Fig. 3:**
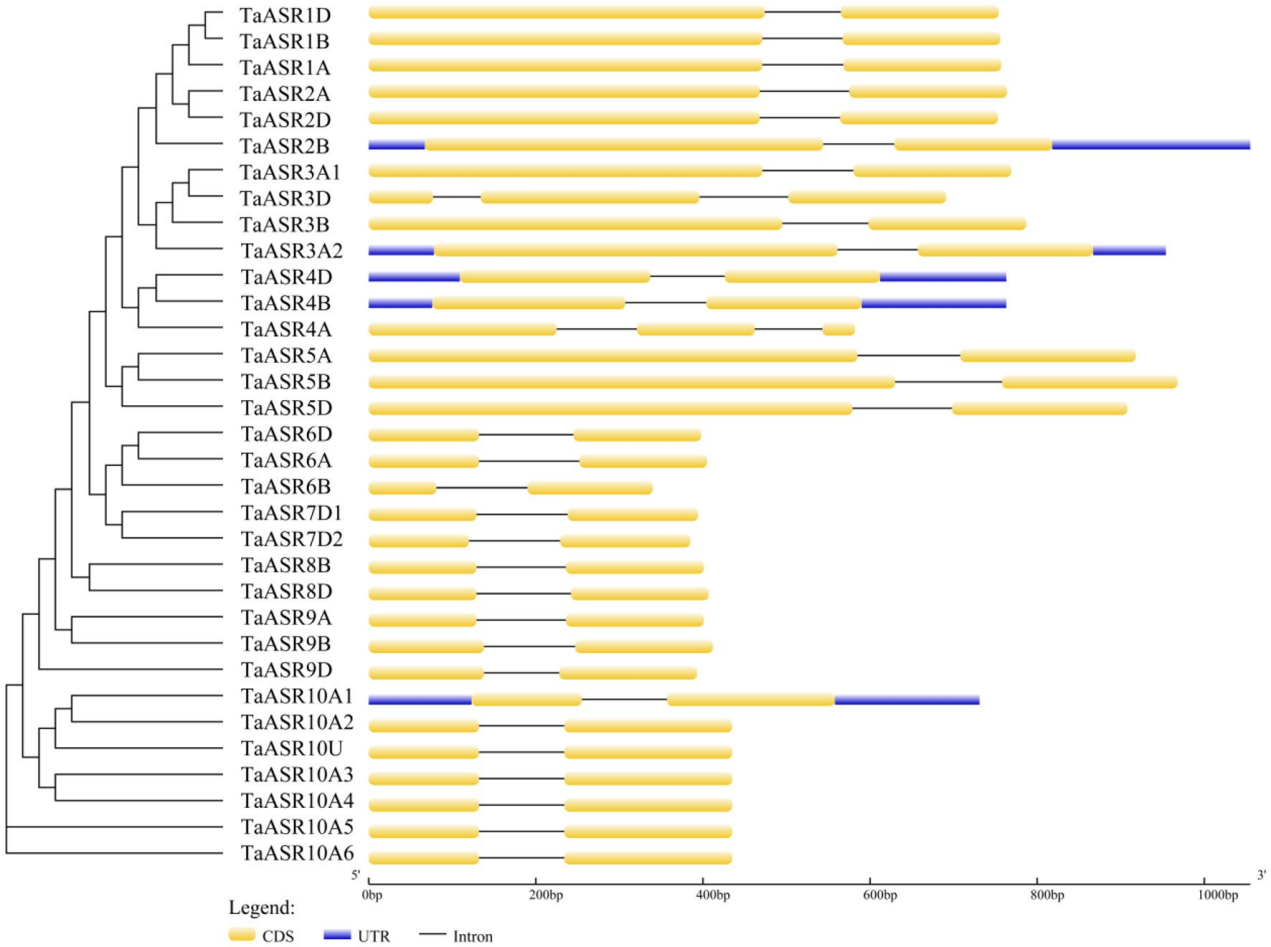
Phylogenetic relationship and gene structure analysis of *TaASR* genes. Phylogenetic tree of 33 wheat ASR proteins (left). The maximum-likelihood phylogenetic tree was constructed using MEGA7.0, with 1000 replicates. Exon–intron structures of *TaASR* genes (right). Yellow boxes represent exons, black lines represent introns, and the upstream/downstream regions of *TaASR* genes are indicated by blue boxes

All the examined 33 *TaASR* genes contained two exons, except *TaASR3D* and *TaASR4A* with three exons (Fig. 3, Additional file 5: Table S4). These results are similar to the *ASR* gene structures of rice and *Brachypodium distachyon* [5, 8]. Genes in the same group are generally more similar in gene structure and lengths of the full gene, intron and exon. Strikingly, all the members in group VI shared the same lengths of the intron and exon, indicating that gene length varied among diverse groups. Group III shared the longest average lengths of the full gene, exon 1, intron 1 and exon 2, while group IV shared the shortest average lengths except intron 1. Among those 33 *TaASRs*, *TaASR2B* and *TaASR6B* were the longest and shortest for the longest UTR and shorter exon 1.

**Fig. 4:**
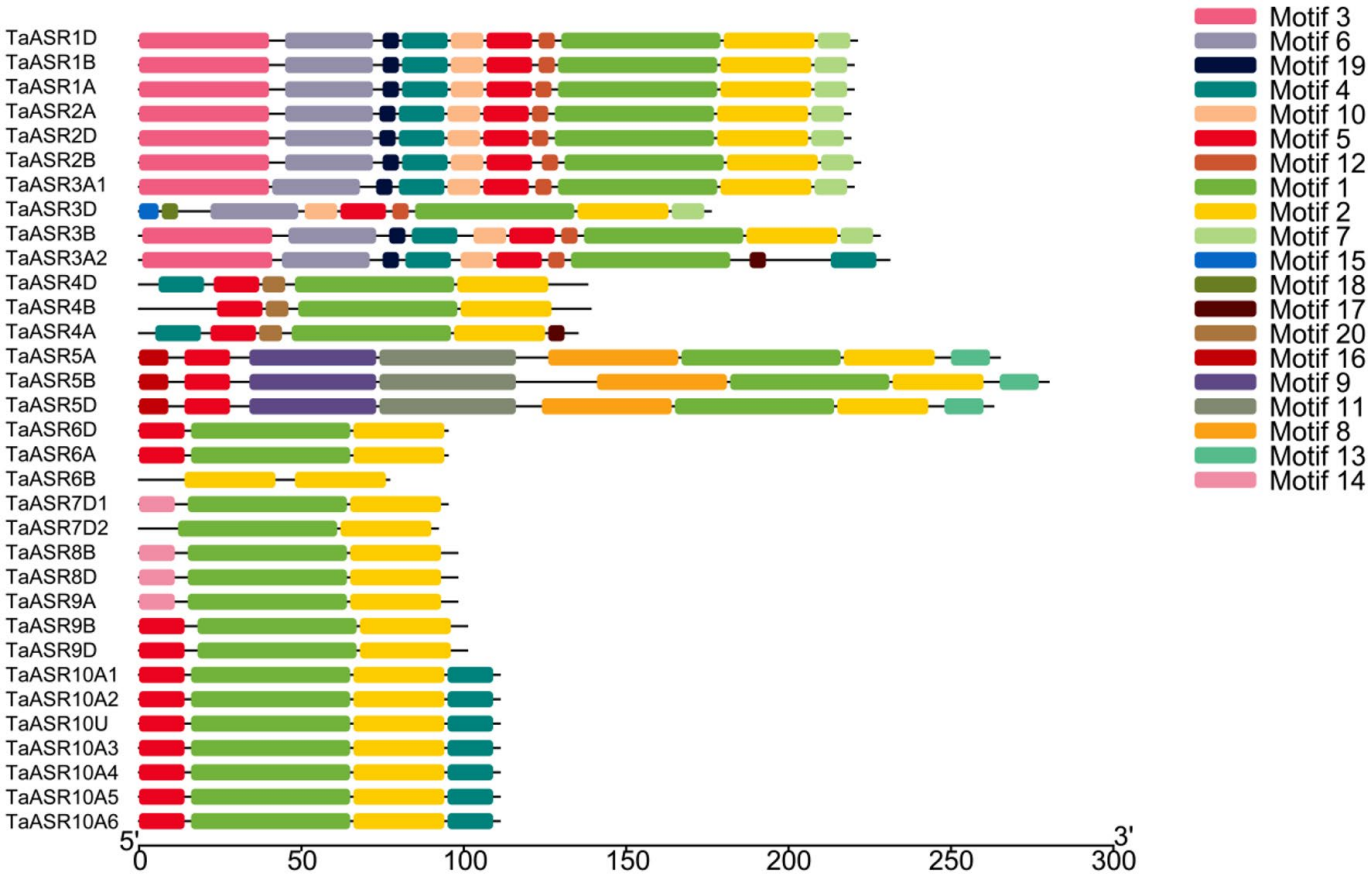
Conserved motifs of 33 wheat ASR proteins. Each colored box represented a different motif. Twenty different motifs are indicated by twenty different colors

Conserved motifs were further predicted using the MEME program. A total of 20 conserved motifs were found in 33 wheat ASR family members (Fig. 4, Additional file 6: Table S5, Additional file 7: Table S6). The identified TaASR motifs varied in length from 6 to 50 aa. Wheat ASRs in the same group shared similar conserved motif composition. For examples, group III and VI shared the same 8 and 4 conserved motifs. Additionally, group I shared the same conserved 10 motifs (except that TaASR3D added motifs 15 and 18 while lacked motifs 3, 4 and 19; TaASR3A2 added motif 17 while lacked motifs 2 and 7). Motifs 1, 2 and 5 existed in all the six groups, except for that one or two motifs were absent from some genes. However, the rest motifs were unevenly distributed among different groups. Motif 4 distributed within group I, II and VI and motif 14 distributed within group IV and V. Motifs 3, 6, 7, 10, 12 and 19 only existed in group I. Motifs 8, 9, 11, 13 and 16 uniquely distributed within group III, while motif 20 was only present at group II. In addition, the motifs were unevenly distributed among the proteins, with the number of motifs ranging from 1 (TaASR6B) to 10 (all group I members except TaASR3D). Motifs 1 and 2 were found in 32 of these ASRs, absent from TaASR6B and TaASR3A2, respectively. Motif 17 was only shared by TaASR3A2 and TaASR4A. It should be noted that motifs 15 and 18 each were uniquely identified in TaASR3D, which might be consistent with its special gene structure.

### Transcription factor binding sites analysis in the TaASR promoters

**Fig. 5:**
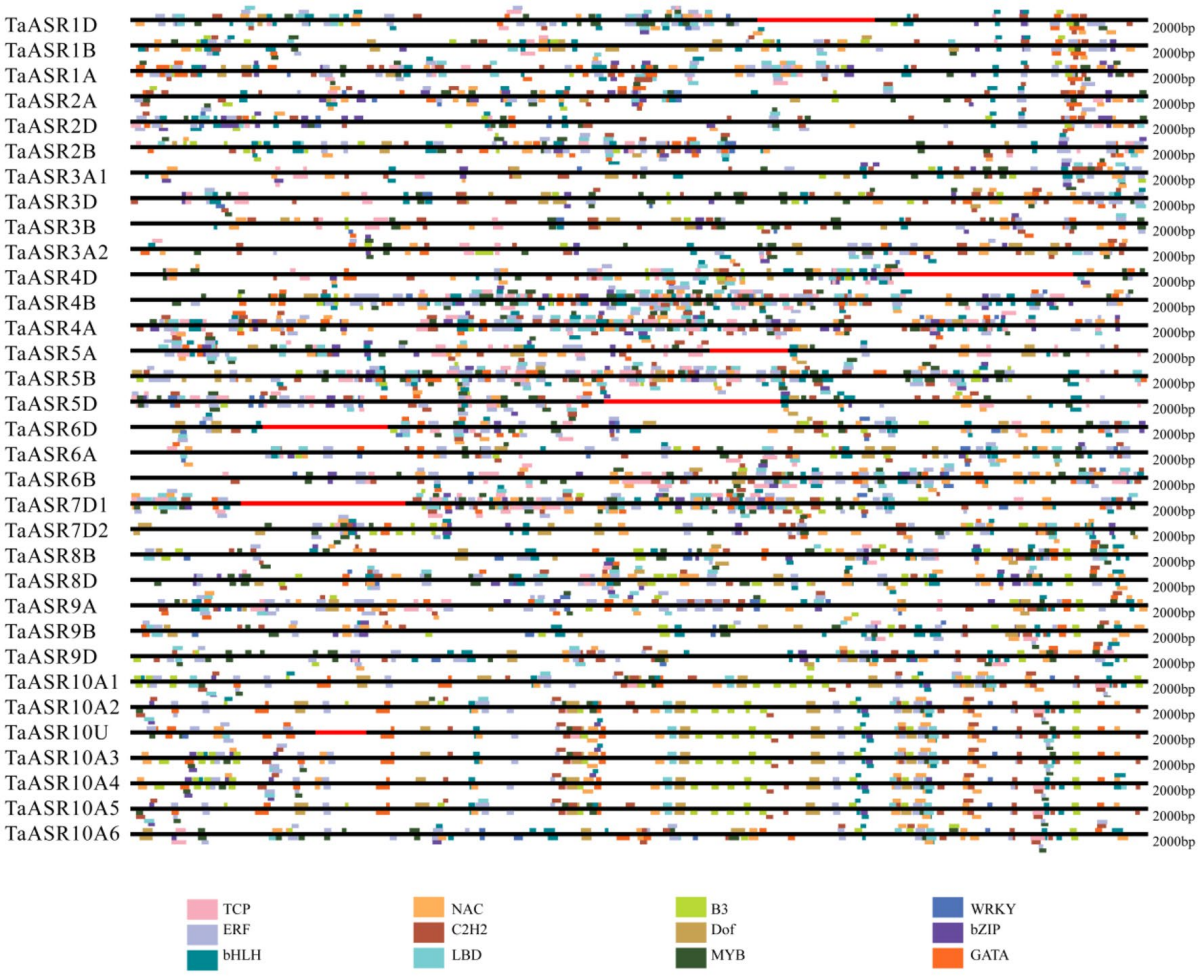
Distribution of transcription factor binding sites in the promoter of *TaASR* genes. Twelve different colors represent twelve different transcription factors

Transcription factors are one of the most regulators that regulate gene expressions at transcriptional level by binding to specific DNA sequences. To explore the possible binding sites of TFs, the 2.0 kb upstream promoter regions of the *TaASRs* were examined via the online database, PlantRegMap. The results showed that a total of 4511 binding cites for 12 TFs including TEOSINTE BRANCHED1/CYCLOIDEA/PROLIFERATING CELL FACTOR1 (TCP) [34, 62], NAM/ATAF/CUC (NAC [63, 64]), B3 [65], WRKY [66], Ethylene Response Factor (ERF) [67], Cys2His2 (C2H2) [68, 69], DNA binding with one finger (Dof) [70], basic leucine zipper (bZIP) [71], basic Helix-Loop-Helix (bHLH) [72, 73], Lateral Organ Boundaries Domain (LBD) [74], myeloblastosis (MYB) [75] and GATA [76–78]were discovered (Fig. 5, Additional file 8: Table S7). ERF, C2H2, bHLH, MYB, NAC, bZIP, LBD, TCP and GATA binding sites occurred 780, 561, 474, 464, 440, 321, 313, 292 and 259 times, respectively, and they were present in the promoter of all 33 *TaASR* genes. 285, 207 and 115 binding sites of B3, Dof and WRKY were identified spanning 32, 32 and 29 *TaASR* promoters, which were absent from 1 (*TaASR5A*), 1 (*TaASR2D*) and 4 (*TaASR1B*, *TaASR5A*, *TaASR5B* and *TaASR7D2*) *TaASRs*, respectively. Most of these TFs, such as NAC [63, 64], WRKY [79, 66], C2H2 [68], bZIP [71], bHLH [72, 73], MYB [75] and GATA [76, 77], were involved in regulating plant growth and development, and the responses to multiple abiotic stress, such as drought and salt. All of the *ASR* genes contained GATA binding sites which were involved in light responsive development [78].

### Expression analysis of TaASR genes in various wheat tissues

**Fig. 6:**
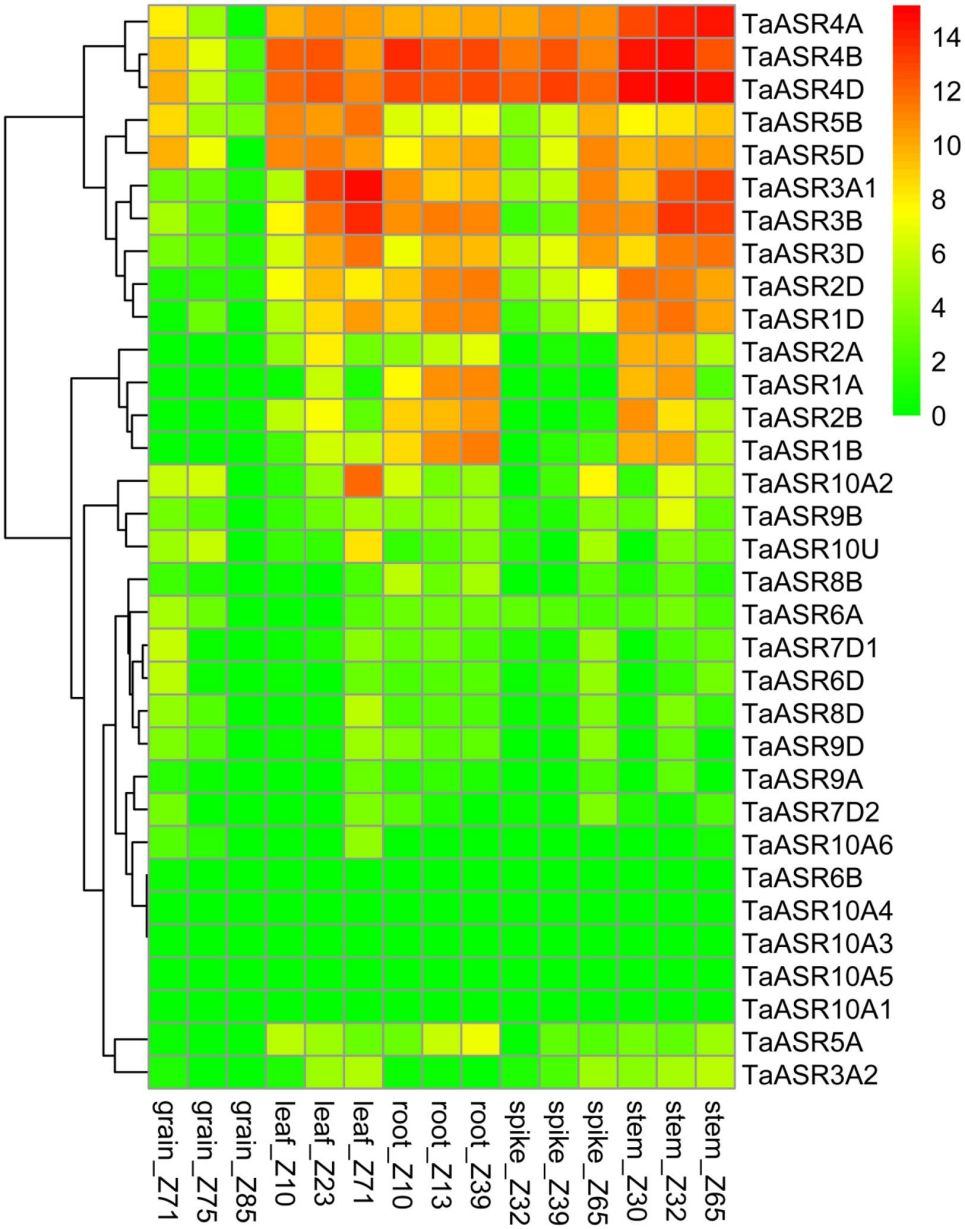
Expression profiles of *TaASR* genes at different developmental stages of five tissues (grain, leaf, root, spike and stem) in wheat

Publicly available RNA-seq databases were used to examine the expression profiles of *TaASR* genes in wheat grain, leaf, root, spike and stem. Results showed that all the examined 33 genes expressed in at least one organ and 24 genes were expressed in all the tested tissues (at least one developmental stage), suggesting *ASR* genes significantly contributed to wheat tissue growth and development (Fig. 6, Additional file 9: Table S8). The expression of group II (*TaASR4D*-*4A*) and group III (*TaASR5A*-*5D*) genes were higher than that of other group genes in multiple tissues overall. They may be involved in the regulation of wheat growth and development. In contrast, most genes in group IV (*TaASR6D*-*7D2*) and V (*TaASR8B*-*9D*) had low or no expression level in almost all tissues, except for *TaASR8B*, *TaASR8D* and *TaASR9B* with relatively high expression level in root_Z10, leaf_Z71 and stem_Z32, respectively. Group I (*TaASR1D*-*3A2*) genes relatively highly expressed in leaf, root and stem, and lowly expressed in grain and spike. Strikingly, three genes (*TaASR1B*-*2A*) did not express in grain. Besides, group VI genes (except *TaASR10A2*-*10U*, *TaASR10A6*) specifically expressed in stem_Z30. *TaASR10A6* lowly expressed in grain, leaf and stem, and did not express in root and spike. Additionally, *TaASR6B* specifically expressed in grain_Z71.

### Expression analysis of TaASRs under abiotic stresses by qRT-PCR

**Fig. 7:**
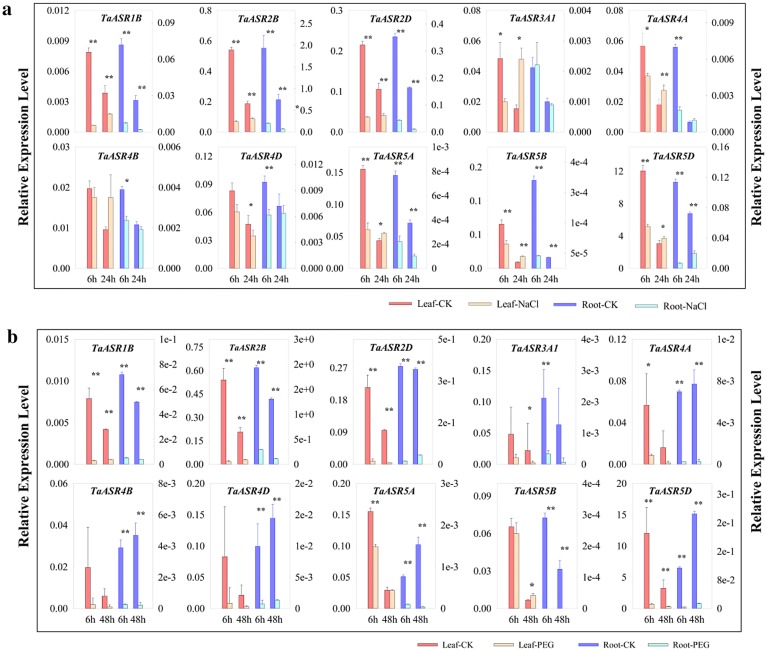
The relative expression levels of *TaASRs*. Expression profiles of 10 *TaASR* genes under NaCl (A) and PEG (B) stress. ^*^and^* *^ indicate *P* ≤ 0.05 and *P* ≤ 0.01, respectively (Student’s *t* test)

The expression patterns of 10 selected *TaASRs* in root and leaf under PEG and NaCl stress were analyzed by qRT-PCR (Fig. 7). The expressions of *TaASR1B*, *TaASR2B* and *TaASR2D *were significantly down-regulated under both PEG and NaCl stresses in root and leaf. In salt stress (NaCl), *TaASR3A1* was down-regulated and up-regulated expressed in leaf at 6 and 24 h, respectively; while remained unchanged in root. In PEG simulated drought stress, the expression of *TaASR3A1* was with no change and significantly increased in leaf at 6 and 48 h, and significantly decreased and with no change in root at 6 and 48 h, respectively. The expression levels of *TaASR4A*, *TaASR4B* and *TaASR4D* either remained no significant change or decreased significantly under both NaCl and PEG stresses, except *TaASR4A* significantly increased under NaCl treatment at 24 h in leaf. Under NaCl treatment, the transcript levels of *TaASR5A*, *TaASR5B* and *TaASR5D* were significantly down-regulated and up-regulated at 6 and 24 h in leaf, respectively; whereas, they were significantly down-regulated in root. Under PEG treatment, they were either down-regulated or not significant changed in leaf, except for that *TaASR5B* was up-regulated at 48 h. In root, they were significantly down-regulated. Taken together, five genes (*TaASR3A1*, *TaASR4A*, *TaASR5A*, *TaASR5B* and *TaASR5D*) were induced in leaf under NaCl treatment at 24 h, while two genes (*TaASR3A1* and *TaASR5B*) were induced in leaf under PEG treatment at 48 h, suggesting these genes might play a vital role in responses to NaCl and PEG stresses in wheat.

## Discussion

Since *ASR* genes were identified in tomato 20 years ago [1], they have been found in various cereal crops. For example, 5, 6, 6 and 10 *ASR* genes were identified and characterized at genome-wide level in *Brachypodium distachyon* [8], foxtail millet [16], rice [5] and maize [7, 9], respectively. In wheat, the information of the *ASR* gene family in genomic scale is still lack. Here, a comprehensive analysis on the *ASR* gene family in the genome-wide level in wheat was conducted.

*ASR* family members have been identified in various plants, including four members in pine [2], five in tomato and *Brachypodium distachyon* [8, 15], six in rice and foxtail millet [5, 16], and ten in maize [9]. In the present study, we identified 33 wheat *ASR* genes, containing a conserved ABA/WDS domain. This suggested that the *ASR* gene family is small, with no more than 33 members and the number of ASR proteins in wheat was much higher than that in other species. It might be attributed to the allohexaploid genome and complex evolution in wheat [80, 81]. Furthermore, the wheat experienced 2 whole genome duplication events from donors of the A, B, and D genomes [45, 82]. Thus, each wheat gene generally has three homologous loci on sub-chromosomes A, B and D [83]. In this study, each of three *TaASR* genes from 6 pairs of homoeologous genes, was found to be on each of the A, B and D homoeologous chromosomes 2, 3 and 4, respectively. Interestingly, there was 1 pair of *TaASRs* with two homoeologous genes (*TaASR8B*, *TaASR8D*) on the homologous chromosomes 3B and 3D. Another 1 pair of *TaASRs* had repeated one time (*TaASR3A1*, *TaASR3A2*) on the homologous chromosomes 3A. This might be caused by the independent evolution and repetitive events between the homologous chromosomes. Gene duplication is generally the main factor causing the expansion of the given gene family [84]. Duplication also allows essential genes to undergo mutations in the duplicated copy, suggesting that similar genes would diverge over the long evolution time period, and improve the expansion and evolution of the gene family [85, 86]. The wheat *ASR* genes in the same group are phylogenetically close to each other rather than with other *ASR* genes from other species including those from monocots, suggesting that they were the product of recent duplication events rather than orthologs of *ASR* genes found in other species. Similar phenomenon was observed in loblolly pine and banana *ASR* genes [3, 87, 88]. In tomato, all four *ASR* genes are located next to each other on chromosome IV and in a tandem array [87]. In the present study, all the *ASR* genes in group VI (except *TaASR10U*) were linked on chromosome 3A within less than 579.8 kb, while *TaASR10U* was located on the unanchored scaffolds. Nevertheless, these *ASRs* from wheat might come from a single gene copy resulted from recent duplication events for the high degree of similarity shared by them in multiple characteristics such as sequence (the same protein sequences), gene structure, chromosomal distribution and phylogeny relationships.

Tandem and segmental duplications of genes have been widely reported for the expansion of different gene families in wheat [89–91]. For examples, 85 tandem or segmental duplications, 22 tandem and 5 segmental duplication events, and 6 tandem and 32 segmental duplication events were identified in *WRKY* [91], *SWEET* [89] and *OPR* [90] gene families in wheat, respectively. Earlier studies have described more frequent tandem duplication events and genes related to stress response have been found to be in distal telomeric segments [92, 93] (IWGSC, 2018). In this study, chromosomal localization revealed that 12, 6 and 8 *TaASR* genes were located on the distal telomeric ends of chromosomes 3A, 3B and 3D, respectively. As respected, 23 of the 26 (except *TaASR3B* and *TaASR9B* on chromosome 3B and *TaASR3D* on chromosome 3D) *TaASR* genes were identified as tandem duplication genes. Furthermore, 24 segmental duplication *TaASR* genes located on chromosomes 2 (A, B, D), 3 (A, B, D) and 4 (A, B, D) were observed in wheat. Tandem and segmental duplication events have also been reported to contribute to the expansion of *ASR* gene family in other species, such as banana [5, 88], tomato [15] and *Brachypodium distachyon* [8]. Additionally, tandem and whole genome duplications also contributed to *ASR* members in rice [5]. Thus, it could be proposed that the tandem and segmental duplication events also contributed to the expansion of the *ASR* gene family in wheat.

In this study, the expression of these segmental duplication *TaASR* genes varied under NaCl and PEG stresses. Group II (*TaASR4D*-*4A*) had different expression patterns in leaf under both NaCl and PEG treatments, and group III only differed in leaf under PEG stress, suggesting the activities of each group genes differentiated after duplication events and they might be functionally important and not redundant. Additionally, *TaASR1B* and *TaASR2B* resulted from a gene duplication event, and had uniform expression patterns under NaCl and PEG treatments; however, these genes had similar expression patterns in five tissues (i.e. grain, leaf, root, spike and stems), suggesting that these genes have similar functions.

Most of the *ASRs* in foxtail millet, maize and rice were ubiquitously expressed in all tested tissues, suggesting the wide functioning of *ASRs* in many development processes in cereal crops [16, 37]. The TF-binding sites analysis suggested that most *TaASR* genes were involved in various processes during growth and development. As expected, most wheat *ASR* genes expressed in multiple tissues and developmental stages, indicating they might play important roles in wheat growth and development. In this study, the expression of group II and III *TaASRs* was generally high in all five tested tissues. The expression of most group IV, V and VI *TaASRs* was low or even no expression existed in almost all tissues. Group II proteins contained six motifs of 1, 2, 4, 5, 17 and 20, of which motif 20 was specific for this group proteins. Group III proteins contained 8 motifs of 1, 2, 5, 8, 9, 11, 13 and 16, of which five motifs (motifs 8, 9, 11, 13 and 16) were specific for this group proteins. Group IV and V proteins contained motifs 1, 2, 5 and 14, while motif 14 was uniquely present in these two group proteins. Group VI proteins contained motif 1, 2, 4 and 5. Therefore, the diverse of expression patterns might be resulted from the diversity of motifs that they contained. Most *TaASRs* in group I had low or no expression in grain and spike. All the group VI *TaASRs* (except *TaASRs*, *10A2*, *10U* and *10A6*) rarely expressed in stem_Z30, while *TaASR6B* only expressed in grain_Z71. Thus, it could be documented that the expression of wheat *ASR* genes exhibit tissue or development stage-specific pattern. These results were similar to *BdASR5*, which expressed at relatively high levels in stem and leaf, while it was not the case in the root in *Brachypodium distachyon* [8]. However, the group I specific motifs of 3, 6, 7, 10, 12, 15, 18 and 19, and motif 2, which was the only motif present in TaASR6B, might contribute to their specific expression patterns. Interestingly, tandem duplication gene pairs *TaASR1A*/*2A*, *TaASR10A1*/*10A5*, *TaASR1B*/*2B*, *TaASR1D/2D*, *TaASR7D1*/*6D* and *TaASR8D*/*9D* respectively shared similar motifs and showed similar expression patterns, which might be regulated by a coordinated regulatory mechanism.

*ASR* genes have been reported to be widely involved in plant responses to various abiotic stresses at the transcriptional level and normally be positively regulated. Overexpression of *OsASR5*, *SiASR1* and wheat *ASR1* enhanced osmotic stress and drought tolerance in transgenic plants [16, 21, 34]. *SiASR4* and *HvASR5*-overexpressing transgenic plants exhibited enhanced tolerance to drought and salt stress [33, 94]. Various binding sites of TFs involved in various stresses regulation, like drought, salt, heat and cold, were found in the promoter regions of *TaASR* genes. Thus, it could be speculated that the wheat *ASR* genes participated in stress responses. The expressions of *TaASR3A1*, *TaASR4A*, *TaASR5A*, *TaASR5B* and *TaASR5D* were up-regulated in leaf under NaCl stress. After exposure to PEG, *TaASR3A1* and *TaASR5B* expression were up-regulated in leaf. Further molecular study of these genes should reveal more functional mechanisms for these genes and contribute to the screening of more candidate genes for contributing to genetic engineering for wheat yield improvement and stress tolerance. Virlouvet et al. [7] reported that PEG decreased *ZmASR5* transcript levels in leaf and *ZmASR2* and *ZmASR7* transcript levels in root. Wang et al. [8] reported that *BdASR4* and *BdASR1* expression levels remained unchanged under PEG and NaCl stresses, respectively; while *BdASR2*-*3* expression levels decreased in exposure to NaCl. In this study, the rest five tested *TaASRs* (*TaASR1B*, *TaASR2B*, *TaASR2D*, *TaASR4B* and *TaASR4D*) expression levels decreased or remained unchanged in leaf and root under NaCl and PEG treatments. Thus, the functions of these *TaASRs* might be regulated by multiple elements, and the present of drought/salt associated TF binding sites might be not directly related to the functioning. Hu et al. [21] proved that the *TaASR1* expression was up-regulated in leaf when exposed to drought/osmotic stress by PEG-6000 treatment; however, in our study, the *TaASR4D* (the same gene as *TaASR1*) transcript levels remained no significantly change in leaf. Wheat varieties, nutrient composition and contents, seedling stage, as well as concentration of PEG-6000 were varied among these studies, and their effects remained inconclusive.

## Conclusion

In summary, our study is the first genome-wide analysis of *ASR* genes in wheat. The chromosomal distribution, phylogenetic relationship, gene structure, composition of conserved motif and TFs binding sites were systematically analyzed. The expansion of the *ASR* gene family in wheat was mainly due to gene duplication including segmental duplication and tandem duplication. The TFs binding sites analysis suggested that most *TaASR* genes were involved in various processes during growth and development as well as stress responses in wheat, which will provide abundant resources for functional characterization of *TaASR* genes. Taken together, our results will provide a more extensive insight on *TaASR* gene family, and also contribute to screen more appropriate candidate genes for further investigation on function characterization of *ASRs* under various stresses.

## Supplementary information

**Additional file 1: Table S1.** Sequences and ID loci information of *ASRs* in wheat and other species.

**Additional file 2: Table S2.** Primers used for qRT-PCR.

**Additional file 3: Figure S1.** Phylogenetic analysis of 33 ASR proteins from wheat.

**Additional file 4: Table S3.** Tandem and segmental duplication gene pairs identified in *TaASRs.*

**Additional file 5: Table S4.** Gene structure of *TaASR* genes.

**Additional file 6: Table S5.** Information of motifs identified from wheat ASR proteins using MEME motif search tool. Note: aa, amino acids.

**Additional file 7: Table S6.** Conserved motifs identified from the *TaASR* genes in wheat.

**Additional file 8: Table S7.** Analysis of TF binding sites in the *TaASR* promoters.

**Additional file 9: Table S8.** FPKM values of wheat *ASRs* in various developmental tissues.

## Data Availability

Please contact author for data requests.
