# Supplementary figures and images for: Genome-wide characterization of the abscisic acid-, stress- and ripening-induced (ASR) gene family in wheat (Triticum aestivum L.)
Source: Biol Res. 2020 May 24;53:23. doi: 10.1186/s40659-020-00291-6 (PMC7247183; doi:10.1186/s40659-020-00291-6)

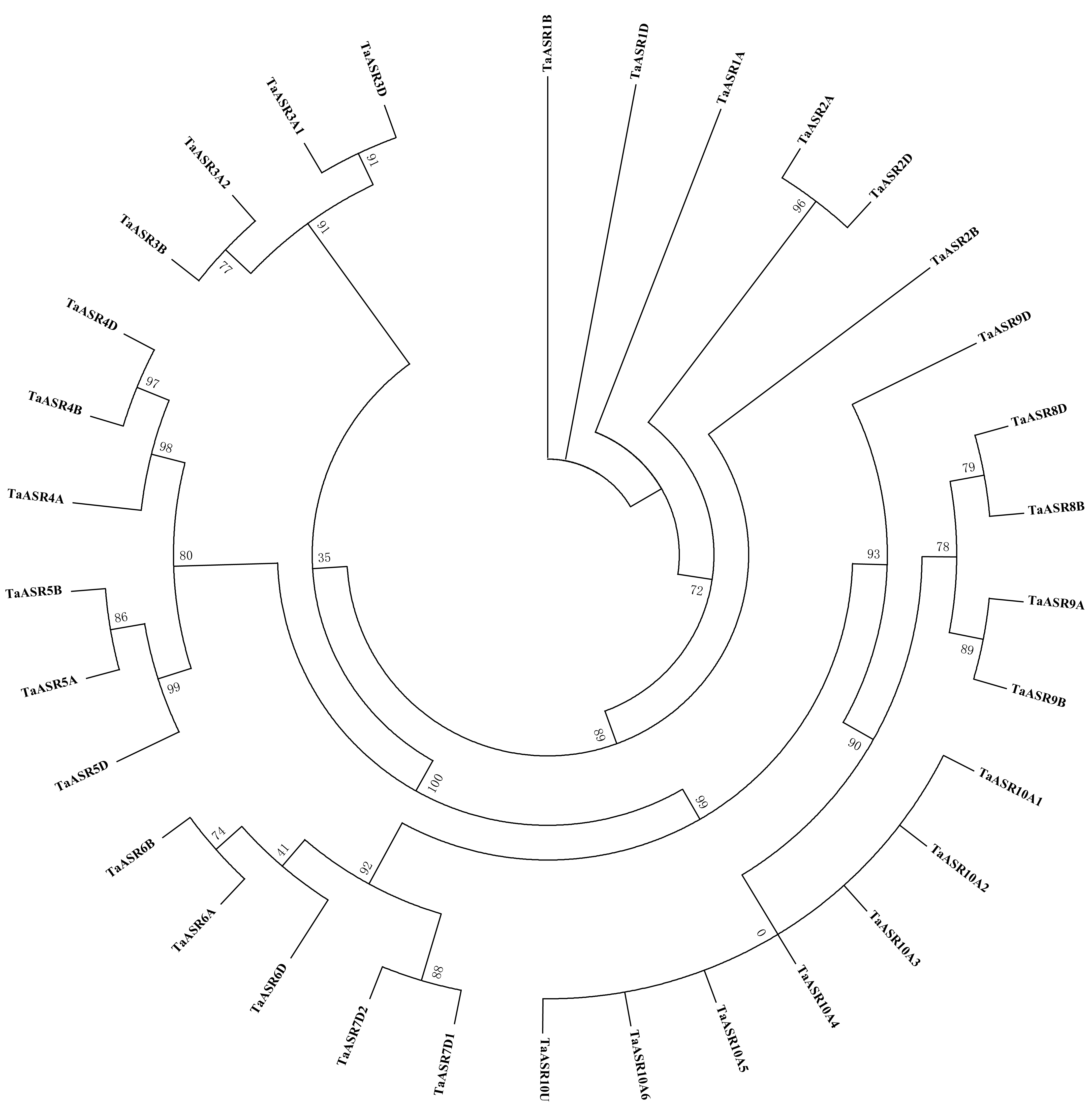

Supplement: Supplementary file 3 — Additional file 3: Figure S1. Phylogenetic analysis of 33 ASR proteins from wheat. [file 40659_2020_291_MOESM3_ESM.tif]
