# Supplementary material for: Genome-wide characterization of the abscisic acid-, stress- and ripening-induced (ASR) gene family in wheat (Triticum aestivum L.)
Source: Biol Res. 2020 May 24;53:23. doi: 10.1186/s40659-020-00291-6 (PMC7247183; doi:10.1186/s40659-020-00291-6)
Supplement: Supplementary file 7 — Additional file 7: Table S6. Conserved motifs identified from the TaASR genes in wheat. [file 40659_2020_291_MOESM7_ESM.docx]

**Table S6 Conserved motifs identified from the TaASR proteins in wheat**

| Motif1 | 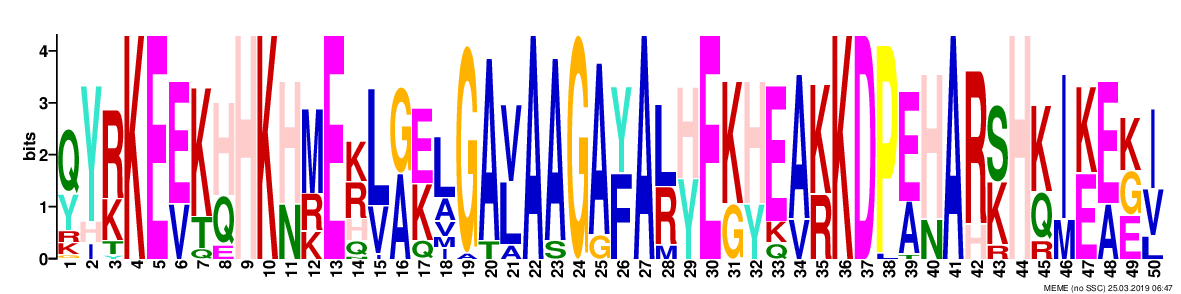 |
| --- | --- |
|  | QYRKEEKHHKHMEKLAELGAVAAGAYALHEKHEAKKDPEHARSHKIEEKI |
| Motif2 | 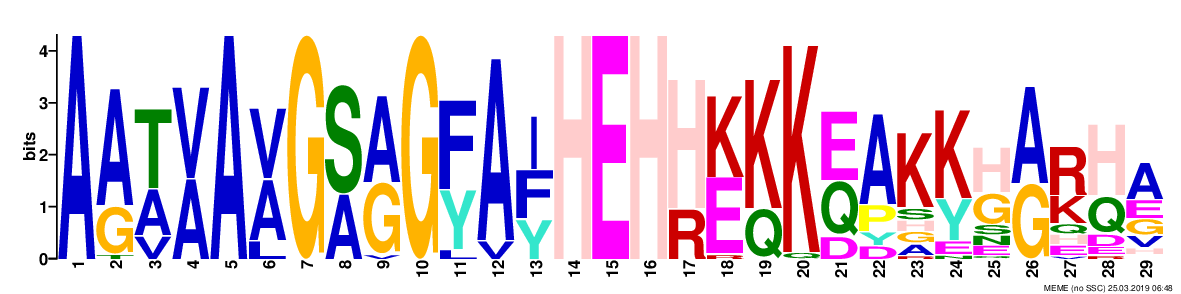 |
|  | AATVAVGSAGFAIHEHHKKKZAKKHARHA |
| Motif3 | 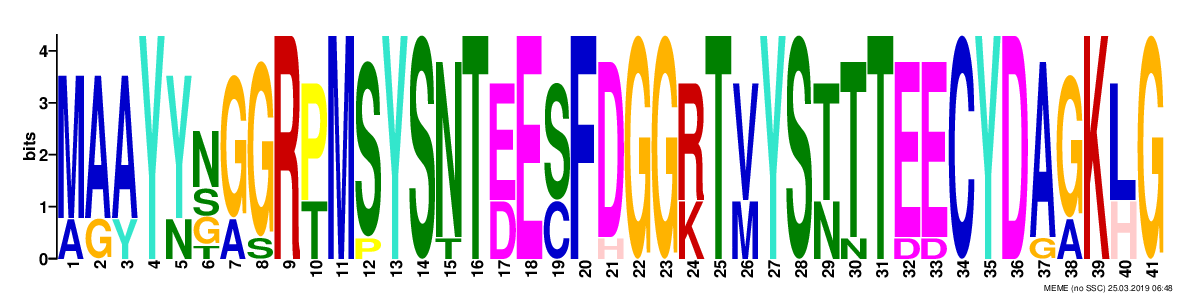 |
|  | MAAYYNGGRPMSYSNTEESFDGGRTVYSTTTEECYDAGKLG |
| Motif4 | 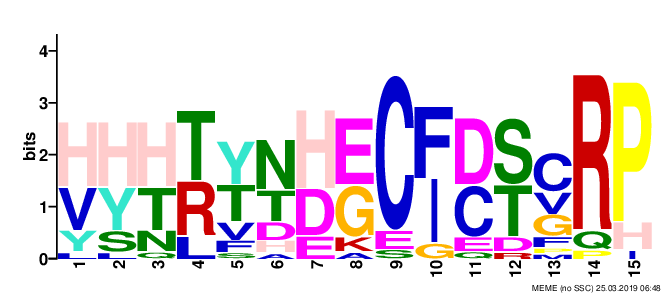 |
|  | HHHTYNHECFDSCRP |
| Motif5 | 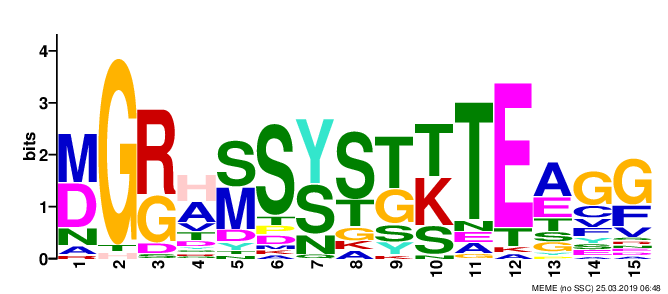 |
|  | MGRHSSYSTTTEAGG |
| Motif6 | 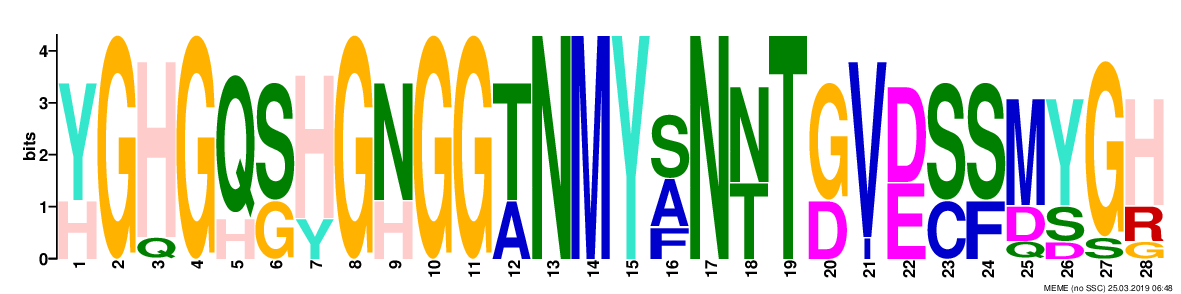 |
|  | YGHGQSHGNGGTNMYSNNTGVDSSMYGH |
| Motif7 | 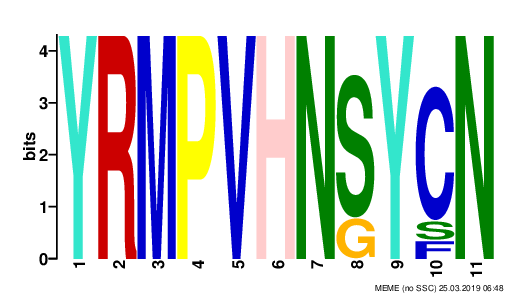 |
|  | YRMPVHNSYCN |
| Motif8 | 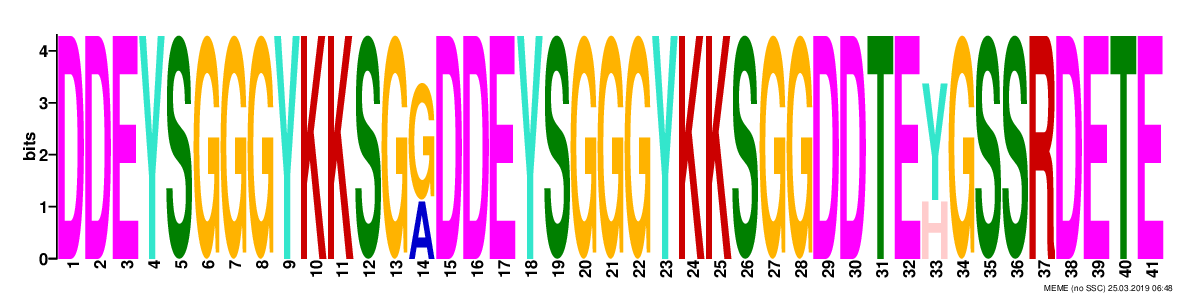 |
|  | DDEYSGGGYKKSGGDDEYSGGGYKKSGGDDTEYGSSRDETE |
| Motif9 | 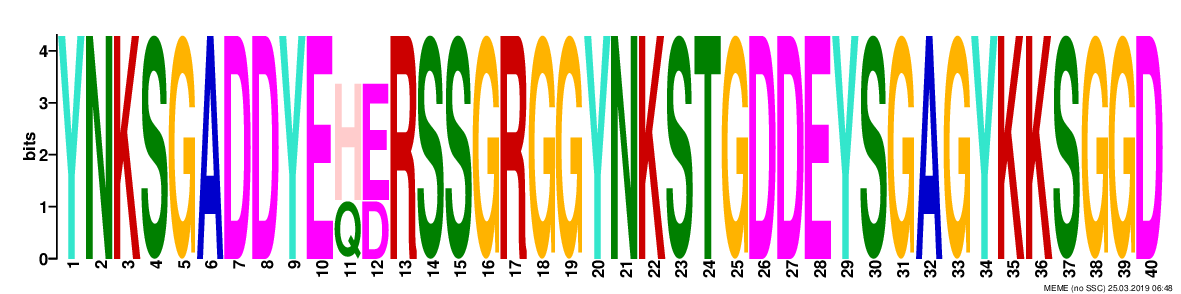 |
|  | YNKSGADDYEHERSSGRGGYNKSTGDDEYSGAGYKKSGGD |
| Motif10 | 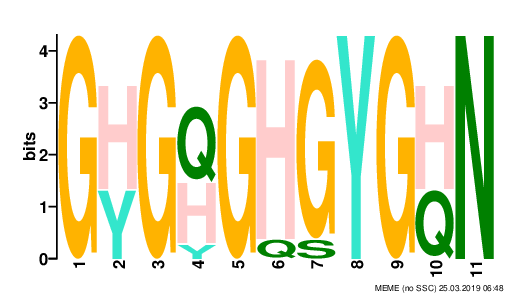 |
|  | GHGHGHGYGHN |
| Motif11 | 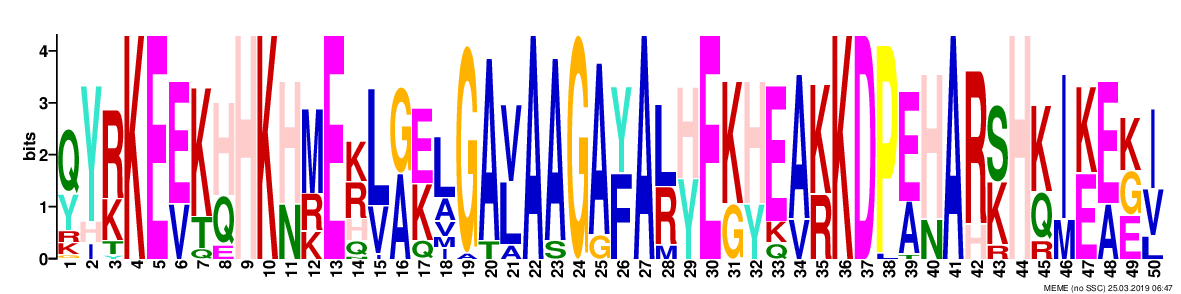 |
|  | DYDTGYNKQSSNEDYGRNKPGSDDYDRSAGGYKKSGGDDDEYS |
| Motif12 | 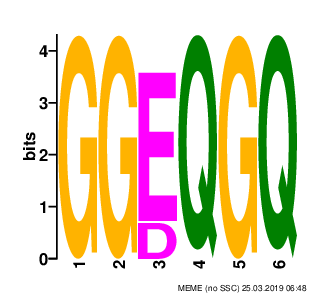 |
|  | GGEQGQ |
| Motif13 | 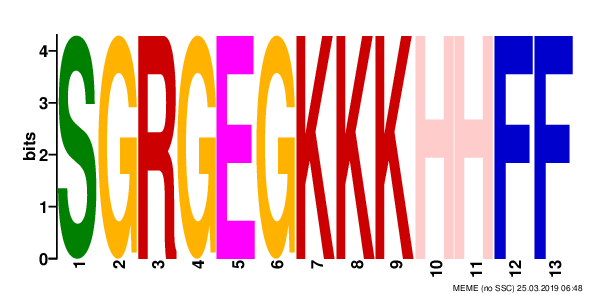 |
|  | SGRGEGKKKHHFF |
| Motif14 | 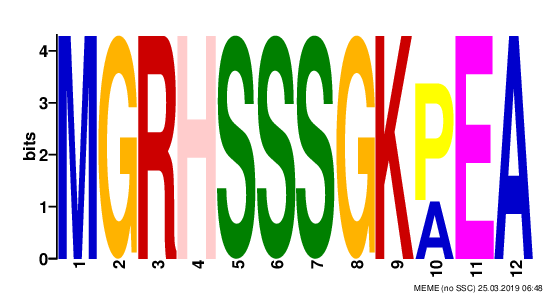 |
|  | MGRHSSSGKPEA |
| Motif15 | 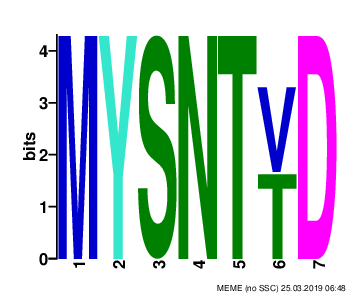 |
|  | MYSNTVD |
| Motif16 | 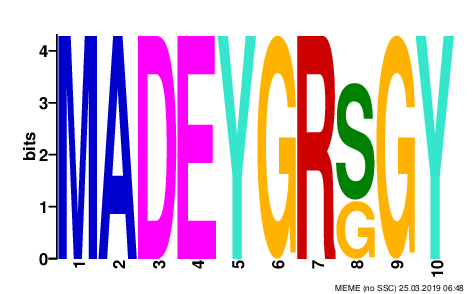 |
|  | MADEYGRSGY |
| Motif17 | 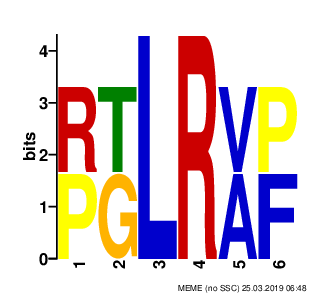 |
|  | RGLRVF |
| Motif18 | 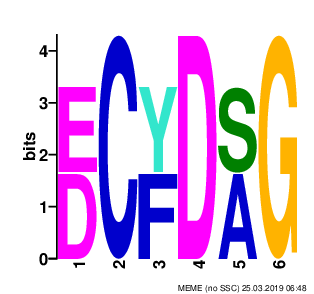 |
|  | ECFDAG |
| Motif19 | 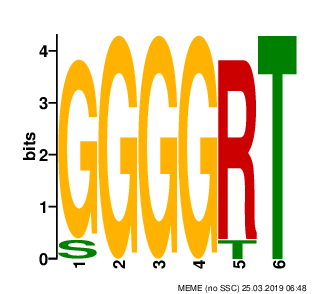 |
|  | GGGGRT |
| Motif20 | 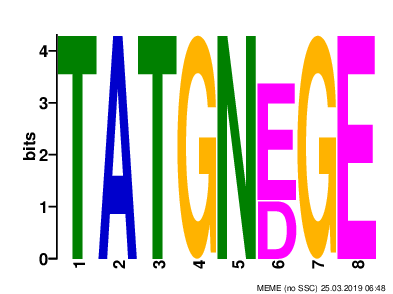 |
|  | TATGNEGE |
